# Cloud-to-Edge Deployment of Optimized nnU-Net for Ischemic Stroke Lesion Segmentation on Resource-Constrained Embedded Devices

**DOI:** 10.3390/s26113322

**Published:** 2026-05-23

**Authors:** Daniel Alcaraz-Ortiz, Juan Francisco Zapata-Pérez, Juan Martinez-Alajarin

**Affiliations:** Escuela Técnica Superior de Ingeniería Industrial, Campus Muralla del Mar, Universidad Politécnica de Cartagena, European University of Technology EUT+, C/Doctor Fleming, s/n, 30202 Cartagena, Spain; juan.zapata@upct.es (J.F.Z.-P.); juanc.martinez@upct.es (J.M.-A.)

**Keywords:** cloud-to-edge deployment, deep learning optimization, edge AI, ischemic stroke, magnetic resonance imaging (MRI), medical image segmentation, nnU-Net v2, NVIDIA Jetson, TensorRT

## Abstract

Ischemic stroke remains a leading cause of global mortality and long-term neurological disability, where the “Time is Brain” paradigm dictates that rapid and accurate lesion assessment is fundamental for effective clinical intervention. While the nnU-Net v2 framework has established a new state of the art in medical image segmentation, its high computational demands and reliance on data-center-grade GPUs hinder its translation into real-time, point-of-care clinical workflows. This study presents a technical feasibility analysis of a Cloud-to-Edge optimization pipeline designed to transfer a 3D nnU-Net v2 model from a high-performance cloud environment to a resource-constrained embedded device. Experimental results showed that edge deployment was associated with a reduction in overlap-based segmentation metrics compared with the cloud reference, with Dice decreasing from approximately 0.78 to 0.67. However, TensorRT FP32 and FP16 inference produced nearly identical mean segmentation metrics, suggesting that reduced-precision inference did not introduce additional measurable degradation under the evaluated conditions. The optimized FP16 configuration achieved a processing time of 10.2 s per 3D volume, representing a 33% reduction compared with embedded FP32 inference, while operating within a low-power envelope of approximately 10–13 W. These findings support the preliminary technical feasibility of executing advanced 3D volumetric segmentation models on low-power edge hardware. Nevertheless, the evaluation was limited to an internal 25-case test subset and did not include external validation, prospective clinical assessment, or reader studies. Therefore, the proposed system should be interpreted as a preliminary deployment framework rather than a clinically validated tool for autonomous stroke imaging.

## 1. Introduction

Ischemic stroke represents one of the most critical medical emergencies worldwide and remains the leading cause of acquired neurological disability in adults [1,2]. The condition is caused by a sudden reduction in cerebral blood flow due to arterial occlusion, initiating a cascade of neuronal damage that progresses rapidly over time. This time dependency is captured by the well-known concept of *Time is Brain.* Saver et al. estimated that approximately 1.9 million neurons are lost every minute during untreated ischemia [3], while delays as short as 15 min can significantly reduce the likelihood of favorable recovery following mechanical thrombectomy [4]. Consequently, international clinical guidelines emphasize minimizing treatment delays to improve patient outcomes [5]. Neuroimaging therefore plays a fundamental role in stroke diagnosis and treatment planning. Magnetic Resonance Imaging (MRI) provides detailed tissue characterization through several complementary sequences, including Diffusion Weighted Imaging (DWI), the Apparent Diffusion Coefficient (ADC) map, and Fluid-Attenuated Inversion Recovery (FLAIR). Diffusion-based sequences (DWI/ADC) are particularly sensitive to acute ischemic injury due to restricted water diffusion, while FLAIR imaging helps identify more evolved lesions. However, manual delineation of ischemic lesions remains time-consuming, requires expert interpretation, and presents considerable inter- and intra-observer variability, limiting its applicability in hyperacute clinical workflows.

To overcome these limitations, deep learning–based medical image segmentation has progressed rapidly during the last decade. The introduction of U-Net provided an encoder–decoder architecture with skip connections capable of preserving spatial information while extracting hierarchical features [6]. Subsequent extensions such as 3D U-Net enabled the direct processing of volumetric medical images, improving segmentation performance in neuroimaging applications [7]. One of the most influential developments in recent years is nnU-Net v2, a self-configuring framework that automatically adapts preprocessing, architecture design, training strategies, and postprocessing to the target dataset [8]. This framework has demonstrated state-of-the-art performance across numerous biomedical segmentation challenges [9]. However, these high levels of accuracy are associated with substantial computational demands, particularly when processing volumetric medical data.

The three-dimensional nature of encoder–decoder networks results in large computational loads due to the number of parameters and the need to store multi-resolution feature maps during inference. As architectures become deeper and more expressive, their computational requirements increase significantly, limiting their deployment in real-time clinical environments or portable devices [10]. Although frameworks such as nnU-Net v2 achieve excellent segmentation accuracy, their execution typically relies on high-performance GPUs available mainly in research laboratories or cloud infrastructures.

In parallel, the emergence of edge computing has opened new possibilities for deploying artificial intelligence systems directly on embedded hardware. Edge AI devices such as the NVIDIA Jetson family are designed to support real-time inference while maintaining low power consumption. These platforms integrate CUDA-enabled GPUs and support hardware-aware optimization tools such as TensorRT, which improves inference performance through reduced-precision computation (FP16), layer fusion, graph optimization, and efficient memory management. Several recent studies have explored Jetson-based solutions in healthcare applications, including dermatological analysis and portable ultrasound systems [11]. Nevertheless, most existing work focuses on lightweight two-dimensional models, while deploying complex three-dimensional architectures on resource-constrained devices remains challenging. Beyond computational efficiency, Edge AI also provides advantages such as reduced latency, improved data privacy, and lower dependence on network connectivity [12,13,14], which are particularly relevant in time-critical scenarios such as stroke diagnosis.

Despite these advances, edge deployment in medical imaging has mainly been explored in lightweight classification, detection, or two-dimensional segmentation scenarios. In contrast, the deployment of high-capacity volumetric segmentation frameworks, such as 3D nnU-Net v2, on resource-constrained embedded hardware remains less extensively characterized. Therefore, the novelty of this work does not lie in the proposal of a new segmentation architecture, but in the systematic evaluation of a complete Cloud-to-Edge deployment pathway for a clinically relevant 3D medical segmentation task.

In this work, we investigate a Cloud-to-Edge workflow for deploying a cloud-trained 3D nnU-Net v2 model on an NVIDIA Jetson Xavier NX device. The main contributions of this work are: (i) the implementation of a reproducible deployment pathway from PyTorch to ONNX and TensorRT; (ii) the evaluation of TensorRT FP32 and FP16 inference on resource-constrained embedded hardware; (iii) the joint analysis of segmentation metrics and hardware-level performance, including latency, throughput, memory usage, and power consumption; and (iv) the identification of the main technical limitations that remain before such systems can be considered for clinically supervised deployment.

The remainder of this manuscript is organized as follows. First, Section 2 reviews previous work on ischemic stroke segmentation and embedded AI. Following this, the materials and methods, including the optimization pipeline and hardware setup, are detailed in Section 3. The experimental results are presented in Section 4, while their clinical and technical implications are analyzed in Section 5. Finally, Section 6 summarizes the main findings and outlines future research directions.

## 2. Related Work

Automatic segmentation of ischemic stroke lesions has evolved rapidly alongside advances in deep learning and hardware-aware computing. In recent years, research has mainly progressed along three complementary directions: (i) development of increasingly accurate segmentation architectures, (ii) benchmark-driven evaluation on standardized datasets, (iii) optimization techniques enabling deployment on resource-constrained hardware.

The ISLES challenge series has played a central role in benchmarking ischemic stroke segmentation methods. Early editions such as ISLES 2015 provided standardized datasets and evaluation protocols that facilitated objective comparison between competing pipelines [15]. Later challenges highlighted the transition from traditional machine learning pipelines to deep learning–based segmentation approaches [16]. More recent datasets, such as ISLES 2022, provide multi-center MRI acquisitions with expert annotations, enabling the development and validation of robust segmentation algorithms under heterogeneous clinical conditions [17,18]. Across these benchmarks, volumetric architectures generally outperform slice-wise approaches in overlap-based metrics, although at the cost of significantly higher computational requirements.

Several neural network architectures have been proposed for ischemic stroke lesion segmentation. Early deep learning approaches such as DeepMedic introduced multi-scale convolutional pathways to capture both local and contextual information in brain lesion analysis [19]. Subsequent developments incorporated residual and densely connected architectures to improve gradient propagation and feature reuse in deeper networks [20,21,22]. More recently, transformer-based models such as TransUNet and nnFormer explored self-attention mechanisms to capture long-range dependencies in medical images, reporting competitive performance across multiple segmentation tasks [23,24]. However, these architectures typically require substantial computational resources and memory capacity, which can hinder their deployment outside high-performance computing environments.

Within this landscape, nnU-Net has emerged as a widely adopted framework for biomedical image segmentation due to its automated configuration strategy and strong benchmark performance [8]. Rather than introducing a new architecture, nnU-Net systematically adapts preprocessing, network configuration, and training strategies to the characteristics of the dataset. This design philosophy has demonstrated strong generalization capabilities across multiple segmentation tasks [9]. Nevertheless, the full-resolution 3D configurations commonly selected for neuroimaging involve large volumetric patches and multi-scale feature maps, resulting in substantial computational and memory demands during inference.

Edge computing has therefore gained increasing attention as a paradigm for deploying AI models closer to the data source. By performing inference directly on embedded devices, edge-based systems can reduce latency, improve data privacy, and decrease dependence on network connectivity [25,26]. In healthcare, Edge AI has been explored for point-of-care diagnostics and distributed clinical systems, including privacy-preserving medical image processing [12] and edge–cloud architectures for the Internet of Medical Things [13]. However, most embedded medical AI studies focus on two-dimensional classification or detection tasks, while volumetric 3D segmentation remains challenging due to memory bandwidth limitations and the large intermediate tensors required during inference.

**Figure 1:**
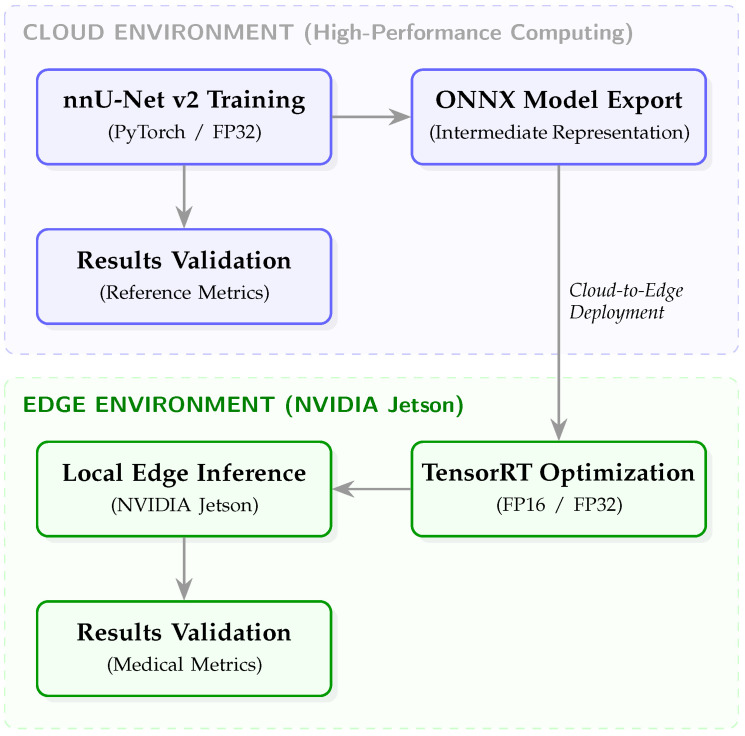
Overview of the proposed Cloud-to-Edge deployment framework for volumetric 3D ischemic stroke lesion segmentation. The pipeline illustrates the transition from high-performance training and ONNX export in the cloud environment to hardware-optimized TensorRT inference and results validation on the NVIDIA Jetson Xavier NX platform.

To enable practical deployment of deep learning models on embedded platforms, several optimization strategies have been proposed. Mixed-precision computation can reduce memory footprint and accelerate inference while maintaining numerical stability when properly calibrated [27]. Post-training quantization enables efficient integer-based inference in many settings [28], and pruning techniques can reduce redundant parameters with limited accuracy degradation [29,30]. Additionally, hardware-aware inference engines can significantly improve execution efficiency by optimizing computational graphs and memory reuse [31]. For example, OpenVINO enables heterogeneous acceleration on CPU-based infrastructures [32], while ONNX-based intermediate representations facilitate interoperability between training frameworks and deployment engines [33]. To overcome the limitations of 3D medical image analysis on resource-constrained hardware and facilitate point-of-care diagnostics, we developed the comprehensive deployment pipeline depicted in Figure 1, which leverages both high-performance cloud training and localized edge optimization. Table 1 summarizes representative approaches in segmenting ischemic stroke lesion, highlighting architectural diversity and the limited adoption of embedded inference platforms.

Despite the progress achieved in both segmentation architectures and inference optimization, comprehensive studies evaluating end-to-end Cloud-to-Edge workflows for volumetric ischemic stroke lesion segmentation remain scarce. In particular, few works combine standardized ISLES benchmarking, automated nnU-Net configuration, ONNX-based model export, TensorRT optimization, and systematic evaluation on embedded platforms such as NVIDIA Jetson devices. This gap is particularly relevant because accurate stroke lesion segmentation could benefit time-critical clinical environments such as mobile stroke units, telemedicine systems, or rural hospitals with limited computational infrastructure. Motivated by this need, the present work investigates a complete Cloud-to-Edge pipeline that integrates high-performance cloud training with optimized deployment on embedded hardware.

## 3. Materials and Methods

This section details the comprehensive methodology employed to design, optimize, and validate the proposed Cloud-to-Edge deployment framework for 3D ischemic stroke lesion segmentation. The study is structured across five key pillars: (i) the characterization of the multi-center ISLES 2022 dataset and the selection of clinical MRI modalities; (ii) the architectural configuration and cloud-based training of the nnU-Net v2 framework; (iii) the multi-stage optimization pipeline used to transform research-grade models into hardware-aware inference engines via ONNX and TensorRT; (iv) the technical specifications and software environment of the embedded NVIDIA Jetson deployment platform; and (v) the dual-evaluation approach encompassing both voxel-level segmentation accuracy and hardware-level performance metrics. By integrating these components, the methodology ensures a rigorous assessment of the transition from high-performance computing environments to resource-constrained clinical edge devices.

### 3.1. Dataset

The experiments conducted in this study are based on the ISLES 2022 dataset, introduced by Hernandez Petzsche et al. [17], which provides a multi-center benchmark for automatic segmentation of acute and sub-acute ischemic stroke lesions from multimodal magnetic resonance imaging (MRI). The dataset was specifically designed to evaluate segmentation algorithms under heterogeneous clinical acquisition conditions and to promote the development of robust and generalizable methods.

ISLES 2022 comprises a total of 400 MRI cases collected from three independent stroke centers: the University Hospital of the Technical University of Munich (Germany), the University Hospital of Bern (Switzerland), and the University Medical Center Hamburg-Eppendorf (Germany). In the official challenge design, the dataset is divided into 250 publicly available training cases and 150 hidden test cases reserved for external challenge evaluation. Since the official hidden test labels are not publicly available, the present study uses only the publicly available training cohort. This cohort was further divided into 200 cases for training, 25 cases for validation, and 25 cases for internal testing, ensuring that the same held-out internal test subset was used consistently for all Cloud and Jetson inference comparisons.

All subjects were adults who underwent MRI as part of routine clinical stroke evaluation. Image acquisition was performed using scanners from multiple vendors and field strengths (1.5 T and 3 T), resulting in considerable variability in spatial resolution and acquisition parameters. In-plane voxel spacing ranges approximately between 0.23 mm and 2.00 mm, while slice thickness varies from 0.68 mm to 9.60 mm depending on center and modality. Differences in repetition time (TR), echo time (TE), and inversion time (TI) further increase inter-site heterogeneity. This variability reflects real-world clinical practice rather than standardized research protocols.

Each case includes three MRI modalities routinely used in hyperacute stroke assessment: (i) Diffusion-Weighted Imaging (DWI), (ii) Apparent Diffusion Coefficient (ADC) maps and (iii) Fluid-Attenuated Inversion Recovery (FLAIR).

DWI highlights regions of restricted diffusion associated with acute ischemic injury. ADC maps provide quantitative confirmation of diffusion restriction and help distinguish true infarction from T2 shine-through effects. Although the original ISLES 2022 dataset also includes Fluid-Attenuated Inversion Recovery (FLAIR) sequences, this study focuses on diffusion imaging (DWI and ADC) due to their higher sensitivity in the hyperacute phase of ischemic stroke. Lesion annotations were generated following a structured multi-stage labeling protocol. After anonymization and format standardization, preliminary lesion masks were produced using a previously trained 3D U-Net model. These initial segmentations were manually corrected by trained annotators, reviewed by neuroradiology residents, and ultimately validated by senior attending neuroradiologists with extensive experience in stroke imaging. In cases where automated pre-segmentation was insufficient, lesions were delineated manually. All annotations were performed by jointly inspecting DWI, ADC, and FLAIR sequences to ensure accurate infarct boundary definition. Lesions were primarily identified as hyperintense regions on DWI with corresponding hypointensity on ADC, consistent with restricted diffusion. FLAIR images were used during the annotation process to provide contextual confirmation and to differentiate acute lesions from chronic findings or imaging artifacts. The final ground-truth masks are binary volumetric segmentations representing infarcted tissue [17]. The dataset encompasses a broad range of infarct sizes, vascular territories, and anatomical locations, including middle cerebral artery (MCA), anterior cerebral artery (ACA), posterior cerebral artery (PCA), and infratentorial strokes. Posterior circulation cases were intentionally represented in higher proportion due to their increased segmentation difficulty. This composition, combined with the inherent multi-center variability, makes the dataset particularly suitable for evaluating models intended for deployment in heterogeneous clinical environments.

### 3.2. Model Architecture and Cloud Training

The segmentation model used in this work is based on the 3D full-resolution configuration of the nnU-Net v2 framework. Rather than relying on manually designed architectures or fixed hyperparameters, nnU-Net v2 analyzes the properties of the dataset and automatically derives an optimized network configuration and training protocol [8]. This design philosophy is particularly advantageous for heterogeneous clinical datasets such as ISLES 2022, where spatial resolution and intensity distributions vary across centers.

Although the original ISLES 2022 dataset provides DWI, ADC, and FLAIR sequences, only Diffusion-Weighted Imaging (DWI) and Apparent Diffusion Coefficient (ADC) maps were used as input modalities in this study. This choice is motivated by the clinical relevance of diffusion imaging in the hyperacute phase of ischemic stroke, where diffusion restriction represents the primary biomarker of infarcted tissue. Acute infarcts typically appear hyperintense in DWI and hypointense in ADC, providing complementary information that facilitates lesion identification. Although FLAIR imaging is available in the dataset, it was not included because FLAIR signal alterations often appear later in stroke evolution and may remain negative in the hyperacute phase. Focusing on DWI and ADC therefore prioritizes the most informative modalities for early infarct detection while reducing input complexity for the segmentation model.

**Figure 2:**
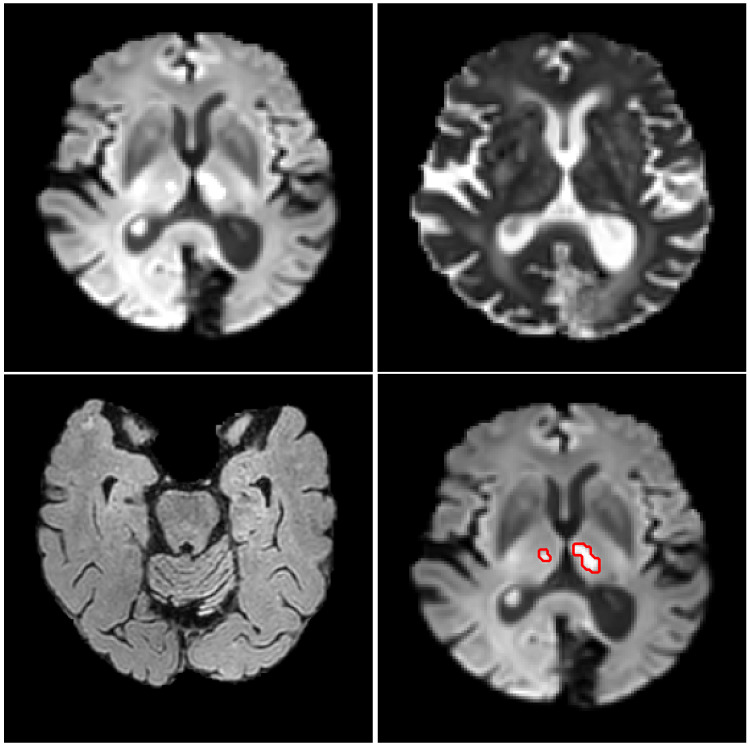
Representative multimodal brain MRI slices from the ISLES 2022 dataset arranged in a 2 × 2 grid. (**Top left**) Diffusion-Weighted Imaging (DWI). (**Top right**) Apparent Diffusion Coefficient (ADC). (**Bottom left**) Fluid-Attenuated Inversion Recovery (FLAIR). (**Bottom right**) DWI slice with the manually annotated ischemic lesion (ground truth) overlaid in red.

To enable multimodal learning, the network was configured with two input channels corresponding to the selected diffusion modalities. Following the nnU-Net v2 data structure convention, each case was stored using a channel-wise indexing scheme (caseXXX_0000 for the DWI volume and caseXXX_0001 for the ADC map). The two volumes were combined into a two-channel 3D input tensor, where DWI was assigned to channel 0 and ADC to channel 1, allowing the model to jointly exploit their complementary diffusion patterns associated with acute ischemic injury. Figure 2 illustrates representative axial slices from the ISLES 2022 dataset, showing the different imaging modalities available in the dataset, including DWI, ADC, and FLAIR, together with the corresponding ground-truth lesion annotation. The model is trained using a subset of these modalities.

All volumes were processed using the preprocessing configuration generated by nnU-Net v2. DWI and ADC volumes were independently normalized using z-score normalization and resampled to an isotropic spacing of 1.0 × 1.0 × 1.0 mm. After foreground cropping and padding when required, the experiment planner selected a full-resolution 3D setup using cubic patches of 128 × 128 × 128 voxels and a batch size of 2.

The fixed 128 × 128 × 128 input size corresponds to the preprocessed nnU-Net space used for training, validation, and controlled deployment experiments. This fixed-shape representation was also used during ONNX export and TensorRT engine generation to ensure compatibility with hardware-specific optimization. Therefore, the same spatial input dimensions were preserved across PyTorch, ONNX, and TensorRT inference.

To avoid introducing preprocessing-related differences between inference environments, all configurations were evaluated using the same preprocessed input tensors and the same postprocessing procedure. Consequently, differences between the cloud FP32 baseline and the embedded TensorRT configurations cannot be attributed to different preprocessing pipelines.

Segmentation labels were handled using a label-preserving procedure. When interpolation generated non-binary intermediate values, the resulting masks were converted back to binary labels using an argmax or fixed-threshold operation before metric computation. Therefore, all reported segmentation metrics were computed on binary volumetric masks.

Although this fixed-shape strategy enabled reproducible TensorRT deployment, it may limit direct applicability to larger or differently cropped full clinical volumes. In particular, the possibility that fixed-shape preprocessing may affect peripheral lesions or unusual lesion locations cannot be fully excluded and is therefore acknowledged as a limitation of the present deployment study.

The resulting architecture follows a six-stage 3D encoder–decoder structure. In the encoder pathway, spatial resolution is progressively reduced through strided 3D convolutions, while the number of feature channels increases to capture increasingly abstract contextual information. The progression of feature maps across resolution levels is [32,64,128,256,320,320], providing sufficient representational capacity without excessive memory growth. Each stage consists of two consecutive 3×3×3 convolutional layers followed by Instance Normalization and LeakyReLU activation. Downsampling is achieved via convolutional strides of 2×2×2 after the first level.

The decoder mirrors the encoder structure, restoring spatial resolution through transposed convolutions and integrating encoder features via skip connections. These skip connections preserve fine-grained spatial information, which is essential for accurate delineation of ischemic lesions that may present irregular and poorly contrasted boundaries. Each decoder level applies two convolutional layers to refine reconstructed feature maps before the final segmentation layer. From the 250 publicly available training cases provided by the ISLES 2022 dataset, a further internal split was performed to allow controlled experimentation. Specifically, 200 cases were used for network training, 25 cases were reserved for validation during optimization, and 25 cases were held out as an internal test subset, as summarized in Table 2. The official ISLES 2022 hidden test set was not used because its ground-truth annotations are not publicly available for independent metric computation. Therefore, the internal 25-case test subset was used to enable a controlled paired comparison between the cloud-based PyTorch FP32 baseline and the embedded TensorRT FP32 and FP16 inference configurations under identical input conditions. These 25 test volumes were never exposed to the model during training or validation and were used as an internal held-out subset for paired comparison between inference configurations. The purpose of this subset was not to replace the official ISLES hidden test set or to establish external clinical generalization, but to ensure that the cloud PyTorch baseline and the embedded TensorRT configurations were evaluated under identical data conditions.

Preprocessing was automatically determined by nnU-Net v2. Both modalities were independently normalized using z-score normalization. Image volumes were resampled using third-order interpolation, while segmentation masks were resampled using first-order interpolation to maintain label consistency. During training, on-the-fly data augmentation was applied, including spatial transformations and intensity perturbations, improving robustness to inter-center variability. Training was conducted in a cloud-based high-performance computing environment equipped with an NVIDIA A100 GPU (40 GB VRAM). The availability of large GPU memory enabled full-resolution 3D training without reducing patch size or network depth. Optimization followed the standard nnU-Net v2 protocol using stochastic gradient descent with Nesterov momentum and a polynomial learning rate decay schedule. A compound Dice and cross-entropy loss function was employed to address class imbalance, as ischemic lesions typically occupy a small fraction of the total brain volume. Training was performed for 120 epochs, after which validation performance plateaued and further improvements became negligible. No manual modifications were introduced to the default nnU-Net v2 3D full-resolution architecture beyond the configuration automatically derived from the dataset fingerprint. The network utilized the standard nnU-Net v2 encoder–decoder design, employing a compound loss function consisting of Dice and cross-entropy. Optimization was performed using Stochastic Gradient Descent (SGD) with Nesterov momentum, a polynomial learning-rate decay schedule, and extensive on-the-fly data augmentation. For the deployment experiments, the model trained on fold-0 was selected as the reference for all inference configurations. The use of a single predefined fold was a deliberate design choice to enable a controlled paired comparison across different hardware and optimization backends. By keeping the trained weights, the fixed 128×128×128 input shape, and the postprocessing procedure strictly identical, we isolated the impact of the Cloud-to-Edge transition from potential variations arising from multi-fold ensemble averaging.

The combined loss function used during training can be expressed as:

(1) $$L_{total}=L_{Dice}+L_{CE}$$

where $L_{Dice}$ denotes the soft Dice loss and $L_{CE}$ represents the voxel-wise cross-entropy loss. The Dice component directly optimizes overlap between predicted and ground-truth segmentations, while the cross-entropy term stabilizes training by providing voxel-level supervision.

During development, nnU-Net v2’s standard cross-validation scheme was available. However, a single predefined split (fold 0) was used in this study to maintain a fixed and reproducible test subset for all deployment configurations. This design allowed paired comparison of PyTorch FP32, TensorRT FP32, and TensorRT FP16 inference on exactly the same cases. Nevertheless, the use of a single split provides a less robust estimate of generalization than full cross-validation and is therefore explicitly acknowledged as a limitation.

This cloud-based training stage ensured that the architecture was fully adapted to the spatial characteristics of the ISLES 2022 dataset while leveraging high-memory GPU resources. The trained network then served as the starting point for the subsequent optimization and deployment steps within the proposed Cloud-to-Edge workflow.

### 3.3. Optimization Pipeline and Cloud-to-Edge Deployment

**Figure 3:**
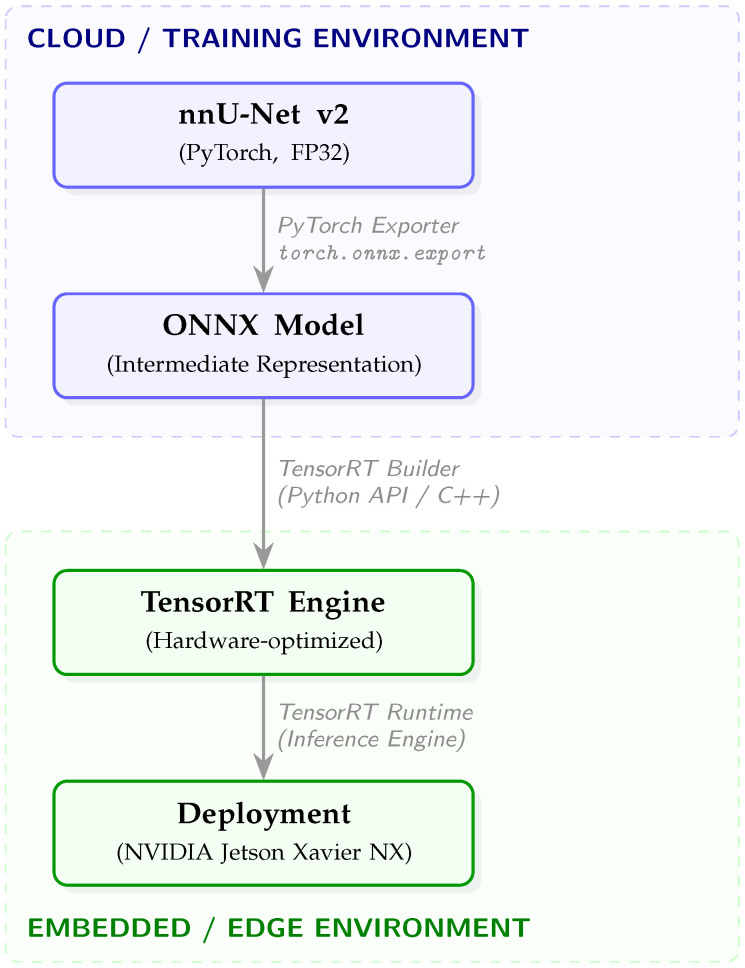
Overview of the embedded/edge deployment environment used in this study. This architecture is particularly suitable for real-time and resource-constrained medical applications. The proposed deep learning model is executed on an NVIDIA Jetson Xavier NX platform, where medical imaging data are processed locally to perform inference. The workflow comprises data input, on-device model execution, and output generation, enabling low-latency responses and eliminating the need for continuous cloud connectivity.

Once the nnU-Net v2 model was fully trained in the cloud environment, the next stage consisted of transforming the research-grade model into a hardware-aware deployment artifact suitable for execution on resource-constrained embedded devices. This transformation required a structured optimization pipeline comprising three main stages: (i) export to an intermediate representation (ONNX), (ii) TensorRT-based engine optimization, and (iii) deployment and validation on NVIDIA Jetson platforms. The overall cloud-to-edge transformation workflow adopted in this study is illustrated in Figure 3.

#### 3.3.1. Model Export to ONNX

The trained PyTorch nnU-Net v2 model was first converted into the Open Neural Network Exchange (ONNX) format using the native PyTorch exporter (torch.onnx.export). ONNX provides a framework-agnostic intermediate representation that decouples model definition from the original deep learning framework, enabling subsequent hardware-specific optimizations. During export, the model input shape was defined using a fixed volumetric size of 128×128×128 voxels, corresponding to the patch size automatically configured by nnU-Net v2 during training. Although ONNX supports dynamic input dimensions, fixed shapes were deliberately used in this work to simplify the deployment pipeline and maximize compatibility with hardware-specific optimizers.

All convolutional layers, normalization operations, activation functions, and skip connections were preserved in the computational graph. Particular attention was given to maintaining numerical consistency between the PyTorch implementation and the exported ONNX graph to avoid discrepancies in inference outputs. After export, the ONNX model was validated through runtime inference checks using representative input volumes, confirming that the exported model produced consistent segmentation outputs and preserved the functional behavior of the original PyTorch implementation. Importantly, the TensorRT inference engine was subsequently built using the same fixed input dimensions (128×128×128). Building the engine for a fixed spatial resolution allows TensorRT to perform more aggressive kernel selection, memory planning, and layer fusion, which significantly improves execution efficiency on resource-constrained embedded hardware such as the NVIDIA Jetson Xavier NX.

#### 3.3.2. TensorRT Optimization

The ONNX (Open Neural Network Exchange, Microsoft Corporation, Redmond, WA, USA) model was subsequently optimized using NVIDIA TensorRT (NVIDIA Corporation, Santa Clara, CA, USA), a high-performance inference engine designed to maximize throughput and minimize latency on NVIDIA GPUs. TensorRT performs graph-level optimizations, kernel auto-tuning, and memory planning to generate an execution engine tailored to the target hardware architecture.

Two precision modes were evaluated on the embedded platform: (i) FP32 (single precision), where the model is executed using 32-bit floating-point arithmetic and TensorRT applies graph optimizations such as layer fusion while preserving full numerical precision; (ii) FP16 (half precision), where model weights and activations are converted to 16-bit floating-point representation to reduce memory footprint and increase throughput by leveraging hardware-specific Tensor Cores.

In both configurations, TensorRT performs automatic layer fusion, combining adjacent convolution, normalization, and activation operations into single optimized kernels. This reduces memory transfers between intermediate feature maps and improves cache utilization. Additionally, TensorRT optimizes memory allocation by precomputing buffer reuse strategies, minimizing dynamic memory overhead during inference. The FP16 configuration further reduces the memory footprint of intermediate tensors and model parameters, enabling faster execution and lower energy consumption. Importantly, since FP16 maintains floating-point representation (unlike integer quantization), segmentation accuracy degradation is typically negligible for medical imaging tasks. The impact of reduced precision is experimentally evaluated in Section 4. Engine building was performed directly on the target Jetson device to ensure that the generated inference engine was fully optimized for the specific GPU architecture, including CUDA cores and Tensor Core availability.

The NVIDIA Jetson Xavier NX (NVIDIA Corporation, Santa Clara, CA, USA) employs a unified memory architecture in which the CPU and GPU share the same 8 GB LPDDR4x memory pool. In this work, explicit manual memory management was not required, as TensorRT internally handles buffer allocation and memory reuse during engine construction and execution. The TensorRT builder automatically plans memory usage for intermediate feature maps and activation tensors, leveraging the unified memory architecture of the Jetson platform to minimize unnecessary data transfers between host and device. This automated memory planning simplifies the deployment pipeline while ensuring efficient utilization of the limited memory resources available on embedded systems.

### 3.4. Hardware Setup

**Figure 4:**
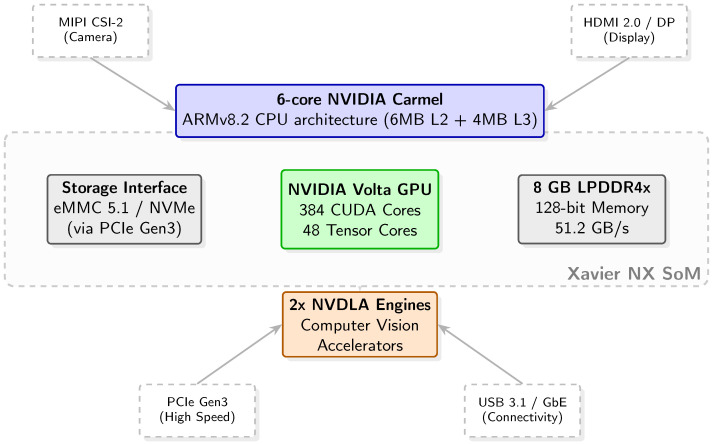
Architectural block diagram of the NVIDIA Jetson Xavier NX System on Module (SoM), highlighting the heterogeneous computing engines and memory subsystem used for edge AI inference.

The embedded inference experiments were conducted primarily on the NVIDIA Jetson Xavier NX Developer Kit, a compact System-on-Module (SoM) designed for edge AI applications requiring a balance between computational performance and energy efficiency. Owing to its high performance-per-watt ratio, the Xavier NX is particularly well suited for portable medical devices and point-of-care diagnostic systems, where computational capability must be balanced with strict power and thermal constraints. The Jetson Xavier NX integrates a 384-core NVIDIA Volta GPU with 48 Tensor Cores, enabling hardware acceleration for both FP32 and FP16 operations. It is equipped with a 6-core NVIDIA Carmel ARMv8.2 64-bit CPU and 8 GB of LPDDR4x memory with a memory bandwidth of 51.2 GB/s. Detailed hardware components and the interconnection bus of the embedded System on Module (SoM) used for the inference experiments are shown in Figure 4.

The device operates within a configurable power envelope of 10 W to 20 W, making it suitable for portable or point-of-care medical scenarios where thermal constraints and energy consumption are critical factors. In contrast to data-center GPUs used during training (e.g., NVIDIA A100), the Xavier NX must manage limited memory resources and reduced computational throughput, which makes hardware-aware optimization essential for volumetric 3D models such as nnU-Net v2. The Volta GPU architecture in the Xavier NX supports mixed-precision computation through Tensor Cores, allowing efficient execution of FP16 operations with minimal accuracy degradation. This capability is particularly relevant in this study, as FP16 TensorRT optimization was leveraged to reduce memory footprint and inference latency while maintaining segmentation fidelity.

For contextual comparison, representative platforms within the NVIDIA Jetson family are summarized in Table 3. The NVIDIA Jetson Nano represents an entry-level platform within the Jetson ecosystem. It integrates a 128-core Maxwell GPU and typically operates within a 5–10 W power range. However, it lacks Tensor Cores and provides limited memory (4 GB), making it significantly less suitable for full-resolution 3D inference of deep volumetric networks. At the higher end of the family, the NVIDIA Jetson Orin series offers substantial performance improvements, incorporating Ampere-based GPUs with a significantly larger number of CUDA cores and Tensor Cores, as well as increased memory capacity. While Orin platforms provide higher throughput and are better suited for computationally demanding AI workloads, their cost and power consumption are correspondingly higher.

The Xavier NX Developer Kit represents an intermediate solution, offering sufficient computational capability to execute optimized 3D nnU-Net v2 inference while maintaining a compact form factor and moderate energy requirements. This balance makes it particularly appropriate for medical edge applications such as mobile stroke units, rural diagnostic systems, or embedded modules integrated into imaging equipment. All inference benchmarks were conducted directly on the Jetson Xavier NX under its maximum performance configuration (20 W, 6-core mode). In this setting, all CPU cores are enabled and GPU frequency scaling is configured to allow sustained peak performance. This configuration provides the highest available computational throughput of the device and was selected to evaluate the upper-bound inference capability of the embedded platform.

The experimental Jetson Xavier NX system was equipped with an NVMe solid-state drive used as the primary storage device for the operating system, model files, and inference scripts. The Jetson Xavier NX Developer Kit used in this study was equipped with the standard active cooling solution provided with the developer kit. The integrated fan was operated under the default automatic control mode of the Jetson platform during the experiments. Active cooling helps mitigate thermal throttling effects during sustained GPU workloads, allowing the device to maintain stable performance during volumetric inference benchmarks.

Although lower power modes (e.g., 10 W or 15 W) are available to reduce energy consumption, they were not considered in this study, as the objective was to assess the feasibility of real-time volumetric inference under the most favorable hardware conditions. The device ran Ubuntu-based JetPack software with CUDA, cuDNN, and TensorRT libraries configured according to NVIDIA’s embedded AI deployment guidelines. Power consumption measurements were obtained using the integrated monitoring utilities provided by the Jetson platform (e.g., tegrastats) to ensure reproducible benchmarking conditions.

To ensure reproducibility of the embedded inference experiments, Table 4 summarizes the key software versions used in the Jetson deployment pipeline.

This software configuration corresponds to the official NVIDIA JetPack distribution for the Jetson Xavier NX and ensures compatibility between CUDA, cuDNN, TensorRT, and the PyTorch runtime. By selecting the Xavier NX as the primary deployment target, this study evaluates the feasibility of executing advanced 3D volumetric segmentation models within realistic embedded constraints, bridging the gap between cloud-based training environments and clinical edge deployment.

### 3.5. Evaluation Metrics

Segmentation performance was quantitatively assessed using a comprehensive set of voxel-level metrics, including Precision, Recall, Accuracy, F1-score, Dice coefficient, Intersection over Union (IoU), and mean Average Precision (mAP). All segmentation metrics were computed by comparing the predicted lesion masks against the corresponding ground-truth annotations across the 25 ISLES 2022 test cases.

To evaluate the spatial agreement between predicted and reference segmentations, two overlap-based metrics widely used in medical image analysis were employed: the Dice Similarity Coefficient (DSC) and the Intersection over Union (IoU). These metrics are defined as:

(2) $$DSC=\frac{2|X\cap Y|}{\left|X|+|Y\right|}$$

(3) $$IoU=\frac{\left|X\cap Y\right|}{\left|X\cup Y\right|}$$

where *X* denotes the predicted lesion mask and *Y* represents the corresponding ground-truth segmentation. The Dice coefficient measures the degree of spatial overlap between predicted and reference regions, while IoU applies a stricter penalization to mismatched areas and therefore typically produces lower values for the same prediction.

Precision, Recall, Accuracy, and F1-score were computed from voxel-wise confusion matrix statistics, while mean Average Precision (mAP) was obtained by treating voxel-wise softmax probabilities as confidence scores and integrating the precision–recall curve across multiple decision thresholds. Beyond segmentation accuracy, computational performance and resource efficiency were evaluated to characterize the feasibility of edge deployment. Throughput was measured as the number of processed volumes per unit time, reflecting real-time applicability in clinical scenarios. GPU memory usage was monitored to quantify the graphics memory footprint during inference, while system RAM usage was recorded to assess overall memory consumption. Power consumption was also measured to evaluate the energy efficiency of the embedded platform, which is particularly relevant for portable or point-of-care medical applications. Together, these metrics provide a holistic assessment encompassing both segmentation accuracy and hardware-level performance across cloud and edge inference environments.

## 4. Results

This section reports the quantitative and qualitative segmentation results obtained under the proposed cloud-to-edge deployment framework. All experiments were performed on the same 25-case subset of the ISLES 2022 test dataset, using identical preprocessed inputs (128 × 128 × 128 voxels, isotropic spacing of 1 mm). The dataset, preprocessing pipeline, and input normalization were kept strictly consistent across all inference configurations to ensure a controlled comparison.

Three inference settings were evaluated: (i) cloud-based inference using PyTorch in FP32 precision; (ii) embedded inference on the NVIDIA Jetson Xavier NX using TensorRT FP32; (iii) embedded inference using TensorRT FP16. All segmentation metrics were computed voxel-wise over complete 3D volumes and subsequently averaged across the 25 test cases.

### 4.1. Segmentation Metrics

The mean segmentation performance metrics and their associated variability for the cloud and embedded configurations are summarized in Table 5. Statistical significance was evaluated using a two-stage non-parametric approach: first, the **Friedman test** was applied to detect global differences across the three inference configurations; subsequently, *post hoc* pairwise comparisons were conducted using the **Wilcoxon signed-rank test** to assess specific performance shifts between the cloud-based reference and the edge-deployed models. Cloud-based inference yielded a Dice coefficient of 0.7784 ± 0.1570, an IoU of 0.6578 ± 0.1697, and a mAP of 0.6402 ± 0.1800. According to the Friedman test, significant global differences were present across all metrics (p<0.001). Post hoc analysis showed that overlap metrics in the embedded Jetson configurations were significantly lower than the cloud reference (p<0.001), with Dice and IoU scores of approximately 0.67 and 0.54, respectively. Precision decreased to 0.7307 ± 0.2391 (p=0.006), while Recall dropped to 0.6576 ± 0.2471 (p=0.018).

**Figure 5:**
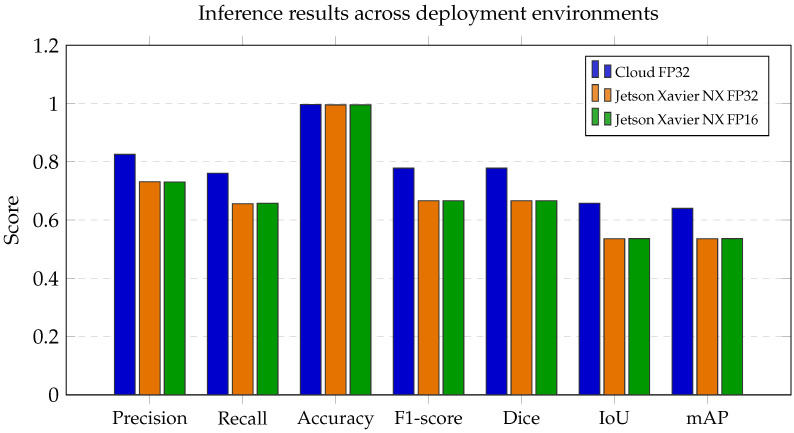
Comparative visualization of mean segmentation performance across the three evaluated inference configurations. Blue bars correspond to cloud-based PyTorch FP32 inference, orange bars represent TensorRT FP32 execution on the NVIDIA Jetson Xavier NX, and green bars indicate TensorRT FP16 execution. Reported metrics include Precision, Recall, Accuracy, F1-score, Dice coefficient, Intersection over Union (IoU), and mean Average Precision (mAP). Values represent averages computed over the 25 ISLES 2022 test cases.

Notably, no statistically significant differences were observed between the Jetson FP32 and Jetson FP16 configurations (p>0.05), confirming that the transition to half-precision inference does not introduce additional measurable degradation beyond the initial cloud-to-edge deployment and fixed-shape optimization pathway(see Figure 5).

### 4.2. Per-Case Distribution Analysis

**Figure 6:**
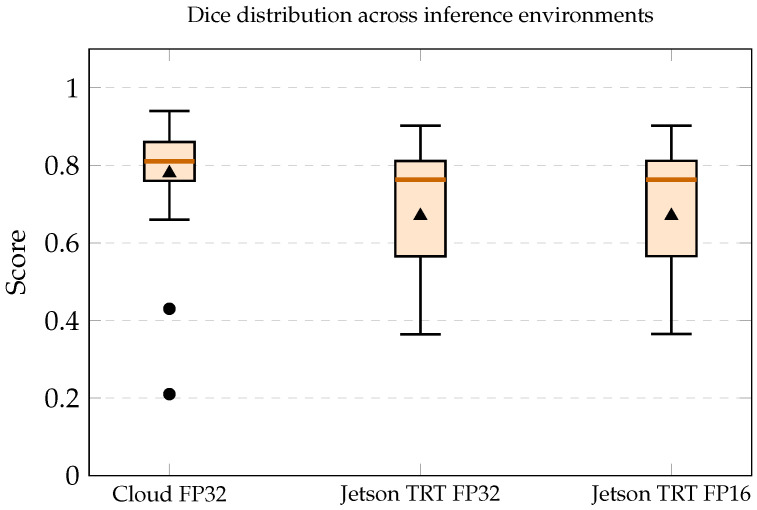
Per-case Dice coefficient distributions across the three evaluated inference configurations. The box represents the interquartile range (IQR), the central orange line indicates the median, and whiskers denote the range excluding outliers. The cloud FP32 configuration shows reduced dispersion and higher median values compared to both embedded TensorRT executions, although a small number of lower outliers are present. Jetson FP32 and FP16 exhibit broader distributions, with nearly overlapping distributions across both precision modes.

To characterize the performance variability across individual test cases, the distribution of Dice coefficients for the three evaluated configurations is presented in Figure 6. The cloud-based FP32 configuration exhibits a relatively compact distribution, with higher median values and a narrower interquartile range compared to the embedded configurations. In contrast, both Jetson FP32 and Jetson FP16 display broader distributions, reflecting increased variability across cases. The embedded configurations also show a moderate shift toward lower Dice values, consistent with the moderately reduced mean performance reported in Table 5. The distributions of Jetson FP32 and Jetson FP16 are nearly overlapping, indicating minimal per-case differences between both precision modes. Compared to the cloud configuration, the edge-deployed models exhibit increased variability. This variability may be related to the combined effects of model conversion, fixed-shape TensorRT execution, numerical differences, and embedded hardware constraints. However, the present experiments do not allow these factors to be fully separated.

Inference latency was measured across the three configurations, as summarized in Table 6. The cloud-based environment achieved the lowest processing time, with a mean of 0.69 s per 3D volume. In comparison, embedded execution on the Jetson Xavier NX resulted in higher latencies, with the FP32 configuration requiring 15.3 s per volume. The application of FP16 precision on the edge device reduced the mean inference time to 10.2 s, representing a 33.0% reduction in latency compared to the embedded FP32 baseline. Specifically, the reported mean inference time of 10.2 s for the FP16 configuration refers to the TensorRT engine execution time plus the necessary GPU memory transfers (Host-to-Device and Device-to-Host). To isolate the computational efficiency of the optimized model, data loading from the NVMe storage and the initial preprocessing stages—such as z-score normalization and resampling—were excluded from this specific latency metric. However, when considering the complete end-to-end processing workflow (including I/O operations and all preprocessing steps), the total execution time remains under 15 s per volume on the embedded platform.

### 4.3. Throughput, Memory and Power Consumption

Hardware performance metrics were recorded during inference on the NVIDIA Jetson Xavier NX. GPU memory utilization, system RAM usage, throughput, and power consumption were monitored using system-level profiling tools. The measured values are summarized in Table 7.

As summarized in Table 7, FP16 execution demonstrated a higher throughput (0.098 volumes/s) compared to the FP32 configuration (0.065 volumes/s). Regarding memory efficiency, the GPU footprint was slightly reduced in FP16 mode (468 MiB) relative to FP32 (480 MiB), while system RAM consumption remained comparable across both configurations. The power draw during inference fluctuated between 12–13W for FP32 and 10–12W for FP16. This observed increase in throughput under half-precision execution is consistent with the reduction in mean inference latency previously reported in Table 6.

### 4.4. 3D Lesion Reconstruction

**Figure 7:**
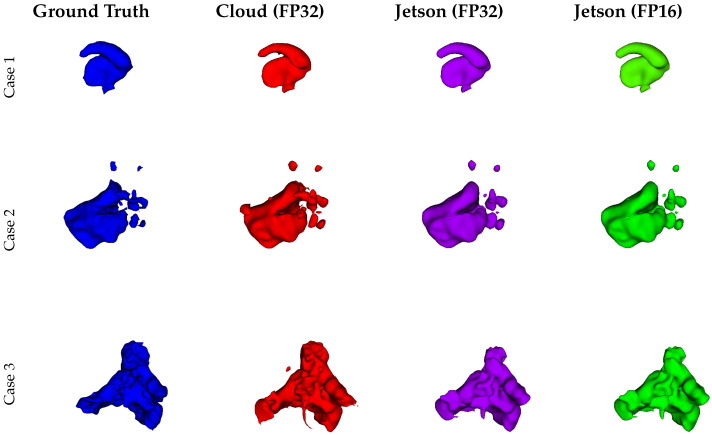
Three representative examples of three-dimensional lesion segmentation across inference configurations. Each row corresponds to a different case from the test set, while columns represent the segmentation source: ground truth annotation (first column), cloud-based nnU-Net inference in FP32 (second column), embedded inference on NVIDIA Jetson Xavier NX using TensorRT FP32 (third column), and embedded inference on NVIDIA Jetson Xavier NX using TensorRT FP16 (fourth column).

In addition to quantitative and hardware performance metrics, qualitative three-dimensional reconstructions were analyzed to visually assess spatial lesion consistency across inference environments. To qualitatively assess volumetric segmentation consistency across inference configurations, three representative cases from the test subset are presented in Figure 7. These examples illustrate different levels of lesion complexity and segmentation agreement between cloud-based and embedded deployments.

As illustrated in Figure 7, the qualitative comparison highlights the consistency of lesion localization across inference environments. (i) Case 1 represents a high-agreement example where all configurations demonstrate similar lesion morphology and spatial localization. (ii) Case 2 illustrates moderate variability across inference configurations while preserving the main lesion structure and spatial distribution. (iii) Case 3 corresponds to a more complex lesion morphology with irregular geometry, where the primary lesion topology is maintained across configurations.

## 5. Discussion

This section evaluates the transition of state-of-the-art volumetric ischemic stroke segmentation from high-performance cloud infrastructures to resource-constrained edge devices. By bridging the gap between theoretical model performance and practical deployment, we analyze the clinical and technical viability of this approach. The following discussion is structured around three critical axes: (i) the morphological and numerical impact of edge deployment on segmentation quality, (ii) the fundamental trade-off between computational throughput and diagnostic reliability, and (iii) the strategic positioning of this decentralized workflow within the evolving landscape of medical AI research.

### 5.1. Segmentation Quality and Morphological Consistency

Experimental results indicate that transferring the nnU-Net segmentation pipeline from the cloud-based PyTorch FP32 reference to the embedded TensorRT configurations leads to a reduction in overlap-based metrics, as shown in Table 8. This decrease is relevant for a medical segmentation task and should not be interpreted as a negligible numerical variation. However, the present experimental design does not fully isolate the individual contribution of each conversion and deployment step. The observed performance drop may result from the combined effect of ONNX export, TensorRT graph transformations, fixed-shape inference, kernel selection, memory-layout changes, numerical differences, and hardware-specific execution on the embedded platform. Therefore, the degradation should not be attributed exclusively to FP16 precision or to the Jetson hardware itself. The comparison between Jetson TensorRT FP32 and Jetson TensorRT FP16 showed very similar mean segmentation metrics, suggesting that reduced precision was not the dominant source of additional degradation under the evaluated conditions. Nevertheless, a complete step-wise ablation including PyTorch FP32, ONNX Runtime FP32, TensorRT FP32 on a workstation GPU, TensorRT FP32 on Jetson, and TensorRT FP16 on Jetson would be required to precisely identify the origin of the performance loss.

A preliminary sub-analysis suggests that the performance drop is more pronounced in cases with smaller lesion volumes (<5 cm^3^), where the fixed-shape resampling (128×128×128) and interpolation artifacts have a higher relative impact on boundary definition compared to the original high-resolution cloud inference.

**Figure 8:**
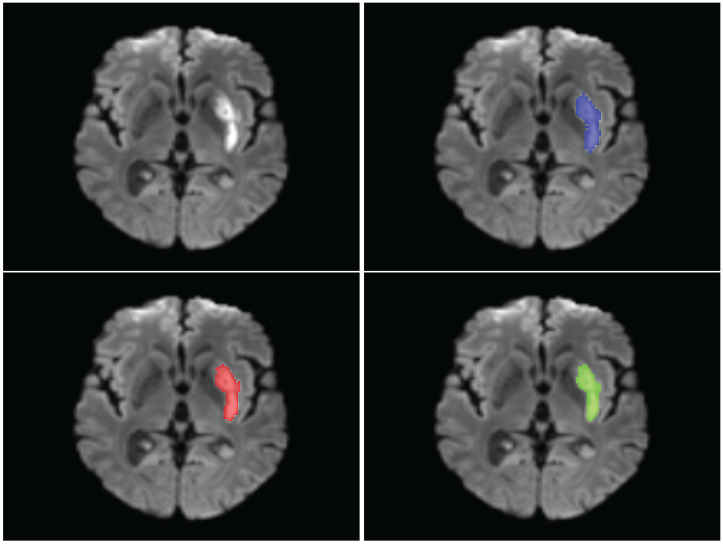
Axial diffusion-weighted imaging (DWI) slice and corresponding segmentation results for a representative case. (**Top left**) Original DWI image. (**Top right**) Expert ground-truth lesion annotation. (**Bottom left**) Lesion segmentation obtained using nnU-Net inference in the cloud (FP32). (**Bottom right**) Lesion segmentation produced by the optimized deployment on the NVIDIA Jetson Xavier NX using TensorRT FP16.

Visual inspection of the volumetric reconstructions supports this interpretation. As illustrated in Figure 7, the principal lesion structures remain consistently localized across all deployment configurations. Differences between the cloud and embedded predictions mainly appear as minor peripheral extensions or surface irregularities. These variations slightly affect the geometric delineation of lesion boundaries but do not alter the global lesion topology. A closer inspection of individual slices provides further insight into this behavior. Figure 8 shows a representative axial diffusion-weighted imaging (DWI) slice together with the corresponding segmentation results obtained from the cloud and embedded deployments. While the main infarct region is consistently identified across configurations, the embedded prediction occasionally exhibits slight peripheral over-segmentation in regions presenting strong DWI hyperintensity.

This phenomenon is likely related to the intrinsic characteristics of diffusion-weighted MRI. DWI sequences are highly sensitive to restricted diffusion but may also exhibit hyperintensities caused by the well-known *T2 shine-through* effect. In clinical practice, radiologists resolve this ambiguity by simultaneously evaluating the corresponding apparent diffusion coefficient (ADC) map. True ischemic infarcts typically appear hyperintense in DWI and hypointense in ADC due to restricted water diffusion caused by cytotoxic edema, whereas regions affected by T2 shine-through remain hyperintense in both modalities. The segmentation model used in this study was trained using both DWI and ADC modalities as input channels, enabling the network to learn this complementary relationship between diffusion-weighted signal intensity and ADC contrast. Nevertheless, minor numerical variations introduced during TensorRT optimization may slightly modify the balance between diffusion-driven intensity features and ADC-based diffusion information, potentially contributing to the peripheral over-segmentation observed in certain embedded predictions.

A particularly relevant observation is the negligible difference between the Jetson FP32 and Jetson FP16 configurations. Across all evaluated metrics, both configurations produce nearly identical segmentation outputs, with Dice differences below 0.001. This negligible impact on segmentation performance when transitioning from FP32 to FP16 suggests that the representational capacity of the neural network is not compromised by the reduced numerical precision. Instead, the observed differences may be influenced by the transformation of the model during the ONNX export and TensorRT compilation stages. During this optimization process, operations such as layer fusion, kernel scheduling adjustments, and mixed-precision execution are applied to maximize computational efficiency on NVIDIA hardware. Although these transformations significantly accelerate inference, they may introduce small numerical variations in intermediate feature activations that propagate through the network and slightly modify the final probability maps used to determine segmentation boundaries.

### 5.2. Hardware Efficiency and Preliminary Deployment Feasibility

A central objective of this work is to evaluate the trade-off between segmentation accuracy and computational efficiency when deploying deep learning models in resource-constrained environments. Cloud-based inference processes each 3D MRI volume in approximately 0.69 s using high-performance GPUs. In contrast, embedded execution on the Jetson Xavier NX requires approximately 15.3 s per volume in FP32 precision and 10.2 s per volume in FP16 precision.

The FP16 configuration therefore provides an approximately 33% reduction in inference time compared to embedded FP32 while maintaining nearly identical segmentation metrics. This behavior reflects the architectural design of modern NVIDIA GPUs, where Tensor Cores are specifically optimized for mixed-precision matrix operations. Consequently, FP16 inference enables substantial acceleration of deep neural network computations without introducing measurable degradation in segmentation performance.

The optimized model running on the Jetson Xavier NX achieved a favorable computational profile, particularly in FP16 precision. However, its potential use in portable imaging platforms or mobile stroke units remains hypothetical at this stage. Real-world clinical integration would require validation on larger multi-center cohorts, assessment of false-negative cases, reader studies, and evaluation of its impact on clinical decision-making and treatment times.

Accordingly, the present results should be interpreted as evidence of preliminary technical feasibility rather than clinical deployment readiness. The main contribution of this work is the demonstration that a high-capacity 3D nnU-Net v2 model can be converted and executed on embedded hardware under realistic memory and power constraints, while preserving a useful level of lesion localization performance. Further methodological refinement and clinical validation are required before considering its use in real-world stroke workflows.

From a translational perspective, the observed reduction in Dice and IoU indicates that embedded deployment still entails a relevant loss of spatial agreement compared with the cloud reference. Therefore, the proposed system should not be interpreted as clinically validated or ready for autonomous decision-making. Instead, the results suggest that embedded inference may be technically feasible for future supervised decision-support workflows, provided that additional validation, failure-case analysis, and prospective clinical evaluation are performed.

Although the Jetson Xavier NX achieved a favorable computational profile, particularly in FP16 precision, its potential use in portable imaging platforms or mobile stroke units remains hypothetical at this stage. Real-world clinical integration would require validation on larger multi-center cohorts, assessment of false-negative cases, reader studies, and evaluation of its impact on clinical decision-making and treatment times. Accordingly, the present results should be interpreted as preliminary technical feasibility evidence rather than clinical deployment readiness.

### 5.3. Comparison with Related Work and Limitations

Most deep learning studies on stroke lesion segmentation report results obtained using workstation-grade GPUs or cloud-based infrastructures. Architectures such as nnU-Net have demonstrated strong segmentation performance across multiple biomedical imaging tasks, but their deployment typically assumes access to high-performance computing resources. In contrast, relatively few studies have investigated the feasibility of executing full volumetric segmentation networks on embedded systems with limited GPU memory and power budgets.

The results presented in this study demonstrate that a complete nnU-Net v2 pipeline can be successfully deployed on an embedded platform equipped with only 8 GB of GPU memory without requiring architectural modifications to the network. Although overlap-based metrics decrease relative to the cloud-based reference implementation, lesion localization and detection sensitivity remain stable. These findings highlight the potential of edge AI systems to enable advanced medical image analysis in environments where access to high-performance computing infrastructure may be limited, including portable imaging devices and mobile emergency units.

Several limitations should nevertheless be acknowledged in the present study. First, the evaluation was conducted on a subset of 25 cases from the ISLES 2022 dataset. Although the dataset provides high-quality expert annotations, larger multi-center datasets would be required to fully assess the generalization capabilities of the proposed pipeline across different imaging protocols and scanner vendors. In addition, the study used a single predefined fold and did not include comparisons with alternative segmentation architectures or baseline edge-oriented solutions. This design was selected to enable a controlled paired comparison between cloud and embedded inference using the same trained model and test cases. However, it provides a less comprehensive assessment than full nnU-Net cross-validation or comparison with DWI-only, DWI+ADC+FLAIR, standard nnU-Net ensemble, or lightweight edge-oriented models.

Second, the volumetric inputs were resampled to a fixed spatial resolution of 128×128×128 voxels in order to comply with the memory constraints of the embedded hardware. While this preprocessing step is commonly applied in deep learning pipelines, it may introduce minor interpolation artifacts that could influence segmentation boundaries. Third, prolonged execution on embedded hardware may lead to thermal throttling under sustained workloads. Although this effect was not observed during the experiments conducted in this study, systematic long-duration stress testing, repeated-run latency variability analysis, and detailed clock-frequency monitoring were not performed. Future evaluations should therefore consider long-term stability under continuous clinical operation. Finally, the optimization pipeline relies on TensorRT compilation, which introduces structural transformations in the original neural network. While these optimizations significantly improve computational efficiency, they may slightly modify the numerical behavior of the model compared to the original PyTorch implementation.

## 6. Conclusions and Future Work

This study establishes the technical feasibility of deploying a state-of-the-art 3D segmentation framework (*nnU-Net v2*) on resource-constrained embedded platforms for acute ischemic stroke assessment. By validating a comprehensive Cloud-to-Edge workflow, this work contributes to bridging the gap between high-performance computing research and the requirements of future point-of-care clinical workflows.

Our results demonstrate that the NVIDIA Jetson Xavier NX can achieve a relevant balance between segmentation sensitivity and computational efficiency. Although a moderate reduction in the Dice coefficient was observed compared to the cloud-based baseline, the achievement of inference times averaging 10.2 s using FP16 precision suggests the potential of embedded AI systems as preliminary technical support tools for rapid stroke image analysis. However, these findings should be interpreted within the scope of a feasibility study, and further validation on larger, prospective, and clinically representative cohorts is required before considering autonomous clinical use. Furthermore, the low power consumption (10–12W) supports its potential integration into portable imaging devices and Mobile Stroke Units (MSUs), where efficient local processing could contribute to future emergency imaging workflows aligned with the “*Time is Brain*” paradigm.

Future research will expand upon these findings in four strategic directions:

(i) Dataset and Robustness: Validating the pipeline on larger, multi-vendor cohorts (e.g., ISLES 2024) to ensure model robustness across diverse acquisition protocols and scanner field strengths.
(ii) Multimodal Integration: Expanding the framework to incorporate CT imaging and perfusion maps, aiming for a more versatile tool for hyperacute neuroimaging decision support.
(iii) Advanced Hardware Optimization: Exploring structured pruning and 8-bit integer quantization (INT8) through TensorRT to further minimize latency while maintaining segmentation fidelity.
(iv) Clinical Validation: Implementing a pilot study in a real-world mobile environment to quantitatively evaluate the system’s impact on door-to-needle times and patient functional recovery.

## Figures and Tables

**Table 1:** Comparative summary of representative deep learning approaches for medical image segmentation and edge deployment, highlighting the gap between cloud-based volumetric frameworks and embedded edge-compatible solutions.

Reference	Model	Architecture Type	Dataset	Platform	3D Support
[19]	DeepMedic	Multi-scale 3D CNN	ISLES/BRATS	Cloud GPU	Yes
[23]	TransUNet	CNN–Transformer hybrid	Multiple datasets	Cloud GPU	Partial
[24]	nnFormer	Transformer-based U-Net	Medical datasets	Cloud GPU	Yes
[8]	nnU-Net	Self-configuring 2D/3D U-Net	MSD/multiple	Cloud GPU	Yes
[11]	CNN-based models	Lightweight 2D CNN	Medical imaging	Edge (Jetson)	No (2D)

**Table 2:** Distribution of the 250 ISLES 2022 cases into training, validation, and test subsets.

Subset	Number of Cases	Percentage (%)
Training	200	80%
Validation	25	10%
Test	25	10%
**Total**	**250**	**100%**

**Table 3:** Comparative analysis of technical specifications and power envelopes for representative NVIDIA Jetson platforms, highlighting the hardware evolution and the trade-off between computational resources (CUDA and Tensor Cores) and energy efficiency for edge AI deployment.

Feature	Jetson Nano	Jetson Xavier NX	Jetson Orin NX
GPU Architecture	Maxwell	Volta	Ampere
CUDA Cores	128	384	1024
Tensor Cores	0	48	32
Memory	4 GB LPDDR4	8 GB LPDDR4x	Up to 16 GB LPDDR5
Typical Power	5–10 W	10–20 W	10–25 W

**Table 4:** Summary of the software stack and development toolkit versions used for the embedded inference experiments to ensure experimental reproducibility.

Component	Version
JetPack SDK	5.1.3 (L4T R35.6.4)
CUDA Toolkit	11.4
cuDNN	8.6
TensorRT	8.5.2.2
PyTorch	2.1 (Jetson build)
ONNX Opset	17
ONNX Runtime GPU	1.16.0

**Table 5:** Quantitative evaluation of segmentation performance. Results are presented as Mean ± Standard Deviation (SD). *p*-values refer to the Wilcoxon signed-rank test comparing Cloud FP32 vs. Jetson FP16 configurations.

Metric	Cloud FP32	Jetson FP32	Jetson FP16	*p*-Value
Dice	0.7784±0.1570	0.6664±0.2323	0.6667±0.2323	<0.001
IoU	0.6578±0.1697	0.5360±0.2214	0.5365±0.2215	<0.001
Precision	0.8256±0.1441	0.7313±0.2389	0.7307±0.2391	0.006
Recall (Sensitivity)	0.7611±0.2008	0.6565±0.2472	0.6576±0.2471	0.018
Accuracy	0.9970±0.0058	0.9956±0.0081	0.9957±0.0081	<0.001
F1-score	0.7784±0.1570	0.6664±0.2323	0.6667±0.2323	<0.001
mAP	0.6402±0.1800	0.5117±0.2274	0.5121±0.2274	<0.001

Statistical significance was assessed using the Friedman test for paired comparisons across the three inference configurations.

**Table 6:** Inference time comparison for the 25 internal test cases. Reported Jetson times correspond to the segmentation stage using pre-built optimized TensorRT engines and exclude cloud training, ONNX export, TensorRT engine compilation, and the original nnU-Net preprocessing stage.

Configuration	Total Time (25 Cases)	Mean Time per Volume(s)
Cloud FP32	17.34 s	0.69 s
Jetson FP32	6 min 22 s	15.3 s
Jetson FP16	4 min 15 s	10.2 s

**Table 7:** Comparative hardware performance and resource utilization during 3D volumetric inference on the NVIDIA Jetson Xavier NX, contrasting TensorRT FP32 and FP16 precision modes.

Metric	TensorRT FP32	TensorRT FP16
Throughput (volumes/s)	0.065	0.098
GPU Memory (MiB)	480	468
RAM Usage (GB)	2.4	2.3
Power Consumption (W)	12–13	10–12

**Table 8:** Comparison of segmentation metrics between the cloud-based nnU-Net inference (FP32) and the embedded deployment on NVIDIA Jetson Xavier NX using TensorRT. Relative change is computed with respect to the cloud baseline. Negative values indicate a percentage decrease in performance compared to the cloud-based reference.

Model	Dice	IoU	Precision	Recall	Accuracy	F1-Score	mAP
ΔFP32 (%)	−14.40	−18.50	−11.41	−13.76	−0.14	−14.40	−16.26
ΔFP16 (%)	−14.35	−18.44	−11.49	−13.60	−0.13	−14.35	−16.20

## Data Availability

The data supporting the findings of this study are not publicly available due to privacy and ethical restrictions related to clinical imaging data.
